# A case of high-flow/high-gradient aortic stenosis due to arteriovenous fistula: how to disarm pitfalls in valvular function assessment

**DOI:** 10.1093/ehjcr/ytag697

**Published:** 2026-09-21

**Authors:** Massimo Mapelli, Valentina Mantegazza, Daniele Poma, Manuela Muratori

**Affiliations:** Centro Cardiologico Monzino, IRCCS, via Carlo Parea 4, Milan 20138, Italy; Centro Cardiologico Monzino, IRCCS, via Carlo Parea 4, Milan 20138, Italy; Centro Cardiologico Monzino, IRCCS, via Carlo Parea 4, Milan 20138, Italy; Centro Cardiologico Monzino, IRCCS, via Carlo Parea 4, Milan 20138, Italy

**Keywords:** Aortic stenosis, High-output heart failure, Arteriovenous fistula, Multidisciplinary approach, High-flow/high-gradient aortic valve, Case report

A 68-year-old man requiring long-term dialysis and kidney transplants was referred in 2021 for aortic valve (AV) surgery due to NYHA class III severe AV stenosis. Then, despite no longer requiring dialysis, he still had a functioning right-arm arteriovenous fistula (AVF) (at the nephrologists’ discretion). B-type natriuretic peptide was elevated (847 pg/mL). Initial transthoracic echocardiography (TTE) showed left ventricular hypertrophy with preserved function, diastolic dysfunction, high AV gradient (peak velocity of 4.4 m/s), and pulmonary hypertension (*Figure 1A* and *B*).

**Figure 1:**
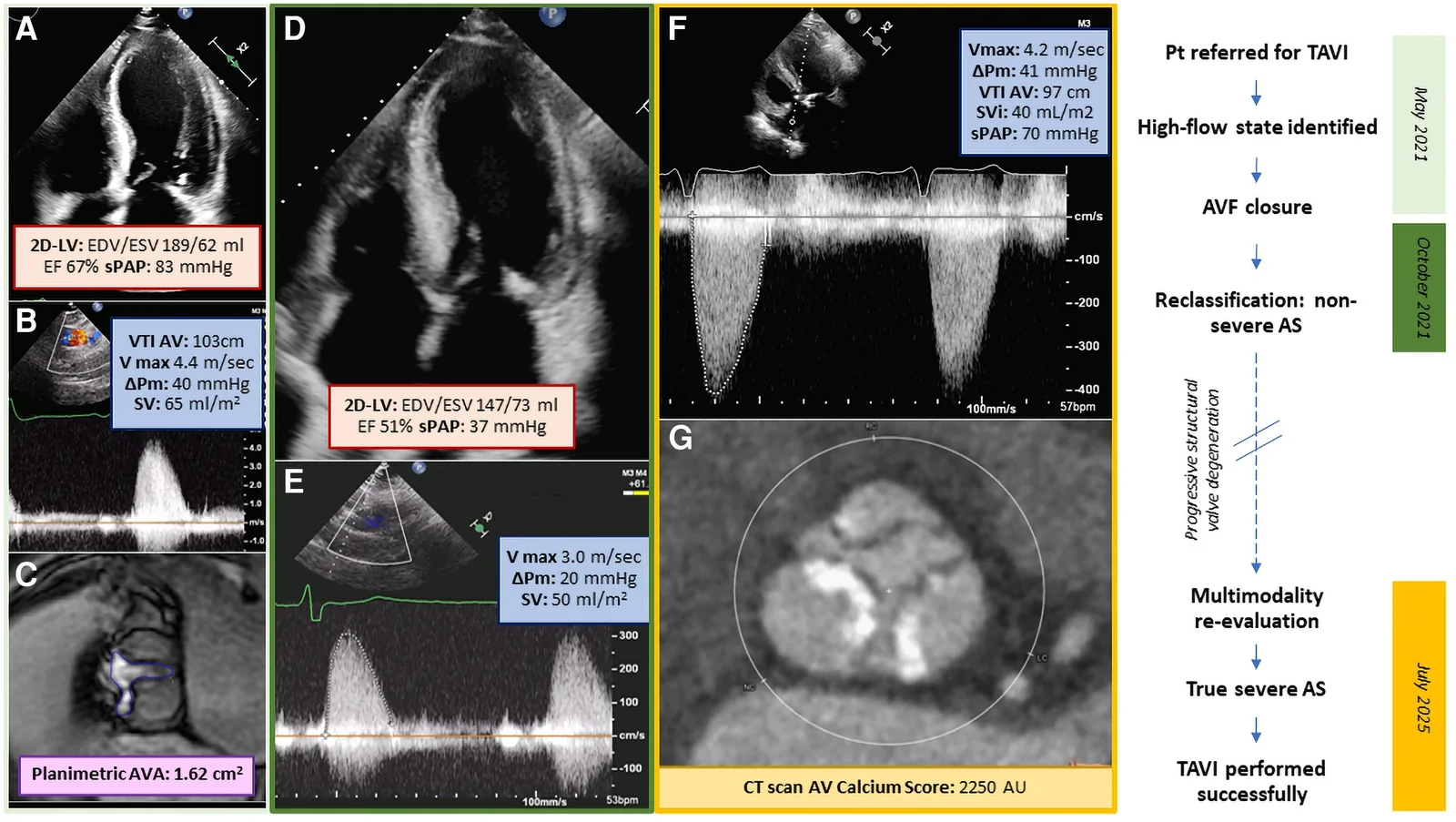
High-flow/high-gradient aortic stenosis: multimodality assessment and longitudinal reclassification. **(*A–C*) (2021 evaluation):** (*A*) Transthoracic echocardiography showing left ventricular dimensions with preserved ejection fraction (EF 67%) and severe pulmonary hypertension (sPAP 83 mmHg) (*A*), alongside high transvalvular gradients (ΔPm 40 mmHg) and an elevated stroke volume index (SVi 65 mL/m^2^) driven by the AVF-associated high-output state (*B*). Cardiac magnetic resonance (CMR) planimetry confirmed a non-severe anatomical aortic valve area (AVA 1.62 cm^2^), establishing the diagnosis of flow-amplified moderate AS (*C*). **(*D*, *E*) (2021: reassessment after AVF closure):** Early post-ligation echocardiography demonstrating the functional downgrade and reclassification to moderate AS. Following the normalization of systemic flow, there is a marked reduction in LV dimensions, transvalvular gradients (*V*_max_ 3 m/s, ΔPm 20 mmHg) and a significant drop in systolic pulmonary artery pressure (sPAP 37 mmHg). **(*F*, *G*) (2025: 4-year follow-up and progression):** Multimodality re-evaluation under normalized flow conditions (SVi 40 mL/m^2^) demonstrating structural progression to true severe AS. Doppler echocardiography reveals recurrent high transvalvular gradients (*V*_max_ 4.2 m/s, ΔPm 41 mmHg) and worsened pulmonary hypertension (sPAP 70 mmHg) (*F*). Computed tomography (CT) confirms severe structural valvular degeneration with a calcium score of 2250 AU (*G*). Abbreviations: AV, aortic valve; AU: Agatston Unit; AVF: arteriovenous fistula; AVA, aortic valve area; AS, aortic stenosis; EDV, end-diastolic volume; EF, ejection fraction; ESV, end-systolic volume; MPG, mean pressure gradient; NYHA, New York Heart Association; sPAP, systolic pulmonary artery pressure; SVi, stroke volume index; TAVI, transcatheter aortic valve implantation; *V*_max_, peak aortic jet velocity; VTI, velocity–time integral.

However, multimodality assessment indicated AV stenosis overestimation in AVF-associated high-flow state [TTE-derived stroke volume index (SVi) 65 mL/m^2^]. Cardiac magnetic resonance (CMR) and total body scintigraphy excluded infiltrative cardiomyopathy (i.e. cardiac amyloidosis), high cardiac output was confirmed by right heart catheterization, also showing narrowed arteriovenous oxygen difference, confirming shunt haemodynamic impact (7.7 L/min; wedge pressure 23 mmHg, mean pulmonary artery pressure 38 mmHg, arteriovenous oxygen difference 32 mL/L). Invasive transvalvular gradients and temporary AVF occlusion were not performed. CMR AV area was 1.62 cm^2^ (*Figure 1C*), and computed tomography (CT)-derived AV calcium score (1612 AU) was incompatible with severe stenosis.

The Heart Team concluded that the gradient was amplified by the AVF-induced high-flow state acting on moderate calcific AS and recommended AVF closure. After ligation of the fistula, TTE documented a significant reduction in AV gradients (*V*_max_ 3 m/s, mean gradient 20 mmHg), normalization of biventricular size, and marked decrease in pulmonary pressure [systolic pulmonary artery pressure (sPAP) 37 mmHg], with parallel clinical improvement, confirming initial AV stenosis overestimation (*Figure 1D* and *E*).

Over a 4-year follow-up, the patient experienced rapid valvular disease progression. While biologically plausible, a causal role for prior high-flow exposure cannot be demonstrated; however, both chronic kidney disease (CKD) and prior high-flow endothelial shear stress may have contributed. By 2025, multimodality reassessment demonstrated severe AS under normal flow conditions (calcium score 2250 AU, *V*_max_ 4.2 m/s, mean gradient 41 mmHg, SVi 40 mL/m^2^) (*Figure 1F* and *G*). The patient underwent successful transcatheter AV implantation.

This case highlights high-flow/high-gradient AV stenosis clinical relevance as potential pitfall leading to premature intervention.^1^ In high-output states, standard echocardiographic parameters can be biased. Specifically, the continuity equation becomes highly sensitive to flow alterations, potentially leading to an inaccurate calculation of the AV area. Direct anatomical evaluation (i.e. CMR planimetry and CT calcium scoring) serve as pivotal complementary assessments, bypassing flow-dependent variables and providing reliable, objective quantification, despite known technical limitations (heavily calcified valves and CKD). Multimodality imaging, invasive haemodynamics, multidisciplinary evaluation, and longitudinal follow-up are key to distinguishing functional from structural severity and optimizing the timing of intervention.^2,3^

## Data Availability

The data underlying this article are not publicly available due to patient privacy and ethical restrictions but are available from the corresponding author upon reasonable request.

## Lead author biography

Massimo Mapelli, MD, PhD, is a cardiologist and Researcher at the Heart Failure Unit of Centro Cardiologico Monzino, IRCCS, and at the University of Milan, Italy. His clinical and research activities focus on heart failure, cardiopulmonary exercise testing, and exercise physiology, with particular interest in the integration of hemodynamic and metabolic responses during exercise. Dr. Mapelli has authored more than 200 peer-reviewed publications and has contributed to several national and international research projects. He is actively involved in mentoring young investigators and promoting translational research bridging clinical cardiology and exercise science.
